# Is It Ethical for Patients with Renal Disease to Purchase Kidneys from the World's Poor?

**DOI:** 10.1371/journal.pmed.0030349

**Published:** 2006-10-24

**Authors:** Tarif Bakdash, Nancy Scheper-Hughes

## Abstract

Background to the debate: In many countries, the number of patients waiting for a kidney transplant is increasing. But there is a widespread and serious shortage of kidneys for transplantation, a shortage that can lead to suffering and death. One approach to tackling the shortage is for a patient with renal disease to buy a kidney from a living donor, who is often in a developing country, a sale that could—in theory at least—help to lift the donor out of poverty. Such kidney sales are almost universally illegal. Proponents of kidney sales argue that since the practice is widespread, it would be safer to formally regulate it, and that society should respect people's autonomous control over their bodies. Critics express concern about the potential for exploitation and coercion of the poor, and about the psychological and physical after-effects on the donors of this illegal kidney trade.

## Abstract 2

There is a worldwide shortage of kidneys for transplantation. Bakdash and Scheper-Hughes debate the ethics of buying a kidney from a living donor.

## Tarif Bakdash's Viewpoint: Poor People Should Have the Right to Exercise Their Autonomy by Selling Their Organs

What would you do if you had to choose between selling your kidney and letting your children starve? I have come to believe that selling one's own kidney would be better than enduring the horrors of poverty. Living below the poverty line on less than a dollar per day makes it hard for parents to feed their children, let alone to clothe them, and organ sales offer a way out of destitution.

After returning to Syria from the United States, I learned about a man who had sold one of his kidneys to help lift his family out of poverty and pay for his children's education. The Arabic news Web site Al-Arabiya (http://www.alarabiya.net) told the story of a young man living in the United Arab Emirates who wanted to sell his kidney in order to help his family of two wives and six children living at their grandparent's home. *The Tribune, India* reported that a 42-year-old Nepalese man named Man Dhoj Tamang sold one of his kidneys to pay off his debts and buy a piece of land [1].

Having trained and taught in North America for almost 14 years, my initial reaction to these reports was that such organ sales were immoral. I was aware that many medical societies and health-care organizations took the position that selling organs is unethical. For example, the Ethics Committee of the Transplantation Society advises transplant surgeons that: “No transplant surgeon/team shall be involved directly or indirectly in the buying or selling of organs/tissues or in any transplant activity aimed at commercial gain to himself/herself or an associated hospital or institute” [2]. The World Health Organization and the International Congress on Transplantation in Developing Countries have also condemned the selling of organs, arguing that it is a coercive practice that exploits the poor [3–5].

Selling one's own kidney would be better than enduring the horrors of poverty.

But then I remembered my own experience of poverty, standing in long lines to buy a few oranges or a little bread, having to live without electricity and running water, and sleeping on the floor with roaches crawling over my face. And then it struck me that *poverty itself* is a kind of coercion. None of the decisions that any poor person makes are made on the basis of free will—instead, these decisions are all dependent on the person's dire financial situation.

We live in a world of startling inequities: of the 10.8 million children under age five who die each year, 10 million (more than 92 percent) live in the lower-income countries [6]. Millions of the world's poorest people have no access to clean water, and no opportunities to educate themselves or their children. It is no wonder that some of these people sell their organs to have a glimpse of a better life. The argument that we should protect the poor from being exploited by banning them from selling their organs is a myth. The poor are always exploited from the day they are born, and in all avenues of life. The only thing of value left for some of them is their bodies.

It is surely a kind of hypocrisy and arrogance on the part of the rich world to reject the right of poor people to exercise their autonomy when it comes to selling their organs. Is it ethically justifiable to deprive the world's poorest people of the chance for a better life? The decision to sell one's organs is never taken lightly—it is often an act of great altruism driven by the desire to create a better life for one's family.

Abdallah Daar, Director of the Program in Applied Ethics and Biotechnology at the University of Toronto, has argued that the position taken by the Ethics Committee of the Transplantation Society “has been totally useless in stopping the increase of the buying and selling of organs” [7]. Unfortunately, those who currently sell their organs risk major complications because the surgery is often done under sub-standard conditions. It would be good medical practice for the buying and selling of organs to be taken out of the black market and become regulated. Instead of banning organ sales, I would add my voice to the growing number of commentators that argue that the sale of organs should be legalized and regulated [8–10]. Janet Radcliffe-Richards and colleagues have argued that “all the evidence we have shows that there is much more scope for exploitation and abuse when a supply of desperately wanted goods is made illegal” [10]. And the best way, they say, to avoid coercion and exploitation of the poor in organ sales would be to ensure “regulation and perhaps a central purchasing system, to provide screening, counselling, reliable payment, insurance, and financial advice.”

Of course we must address the underlying root causes of poverty, so that people are never forced to have to sell their bodily organs. But until we solve the problems of social, political, educational, and economical underdevelopment we need to face reality by legalizing and regulating organ sales in the developing world.

### Acknowledgments

I would like to thank Peter Singer, Program Director of the Master in Health Science in Bioethics, for his guidance and teaching. The views in this article are the author's alone.

## Nancy Scheper-Hughes' Viewpoint: Dividing the World into Organ Buyers and Sellers Is a Medical, Social, and Moral Tragedy

The late Michael Friedlaender, a transplant nephrologist at Hadassah Hospital in Jerusalem, was initially “adamant that organ trading was wrong and would lead to terrible crimes” [11], but he later changed his position. He described how 300 of his patients, Jews and Arabs, traveled abroad for illegal kidney transplants from paid living donors. Although a few of these patients fell seriously ill and one died as a result of their illicit “black market” transplant, most fared as well or better than those transplanted safely at home with a cadaver kidney. Friedlaender joined several respected medical colleagues and prominent bioethicists in supporting proposed legislation in Israel to govern regulated kidney sales [12]. Their refusal to condemn the kidney trade helped pave the way for a global kidney trade that harmed as well as healed people and that engendered new antagonisms toward Israel as a global leader in “transplant tourism.” In August 2006, the Jerusalem district court instructed HMOs to pay kidney donors US$14,000 to cover their expenses, essentially legalizing sales [13]. The philosopher Janet Radcliffe-Richards and her colleagues also called for a regulated market in organ sales: “If a living donor can do without an organ,” they said, “why shouldn't the donor profit and medical science benefit?” [10].

When it comes to organ sales, the ethical conflict between the principles of non-malfeasance (“do no harm”) and beneficence (the moral duty to perform good acts) is being resolved via the market principle: those able to broker or buy a human organ should be allowed to do so. Paying for a kidney “donation” is often described as a “win–win” situation beneficial to both parties [10]. Patient autonomy has become the final arbiter of medical values. Social justice and notions of the good society hardly figure in these discussions. Virtue in suffering and grace in dying can only appear as patently absurd.

Putting a market price on body parts exploits the desperation of the poor.

But the transformation of a person into a “life” that must be prolonged or saved at any cost has turned human life into the ultimate commodity fetish. The absolute value of a single human life saved or prolonged *at any cost* ends all ethical inquiry and erases any possibility of a global social ethic. Meanwhile, the traffic in kidneys reduces the human content of all the lives it touches.

The arguments for “regulation” as opposed to prohibition have some merit, but are out of touch with the social and medical realities in many developing countries. Often institutions in these countries created to “monitor” organ harvesting and distribution are weak, dysfunctional, corrupt, or compromised by the impunity of the organ brokers, and by outlaw surgeons willing to violate the first premise of classical medical bioethics: above all, do no harm.

The results of the few available studies of the effects of nephrectomy on kidney sellers in India [14] and Iran [15,16] are clear. Even under attempts (as in Iran) to regulate and control systems of “compensated gifting” by the Ministry of Health, the outcomes are troubling. Paid donors are not followed and some who encounter subsequent medical problems are turned away. Our research among hundreds of kidney sellers in Moldova, Romania, Turkey, the Philippines, and Brazil has shown that many suffer post-operatively from chronic pain, social isolation, stigma, and severe psychological problems [17]. Their economic conditions decline following the sale due to negative perceptions and self-perceptions of kidney sellers as weak and disabled individuals. The feelings of disappointment, anger, resentment, and even seething hatred by kidney sellers toward the surgeons and the recipients of their organs suggest that the practice engenders deep social pathologies. These outcomes have been found in countries where kidney selling is illegal as well as in Iran, where kidney selling is legal and regulated.

Organs Watch, an independent, university-based human rights and research project, has provided assistance to kidney sellers in Moldova, Brazil, and the Philippines, including diagnostic exams and sonograms. These revealed that many organ sellers face a range of post-operative complications and medical problems, including hypertension and kidney insufficiency, without access to adequate medical care or medications (http://sunsite.berkeley.edu/biotech/organswatch/pages/research.html). Kidney sellers find themselves unemployable because they are unable to sustain the demands of heavy agricultural or construction work, the only labor available to men with their skills. Kidney sellers are often alienated from their families and coworkers, excommunicated from their churches, and excluded from marriage. The children and spouses of kidney sellers are subject to cruel taunts (“Your father is a one-kidney!”) and ridicule.

In our studies, male kidney sellers suffered from exclusion by potential employers and coworkers, and by girlfriends and wives who labeled them as “weak,” “inadequate,” or mutilated. “No young woman in the village will marry a man with the tell-tale scar of a kidney seller,” a village elder in Mingir, Moldova, told me. Even in the United States, kidney donors have died or become comatose as a result of donation [18]. In the context of for-profit transplant tourism, nephrectomy is a risky procedure [19].

The violence associated with kidney selling gives reason to pause.

Bioethical arguments supporting the right to sell an organ are based on Euro-American notions of contract and individual “choice.” But the social and economic contexts make the “choice” to sell a kidney in an urban slum of Calcutta, or in a Brazilian *favela* or Philippine shantytown, anything but a “free” and “autonomous” one. Consent is problematic with “the executioner”—whether on death row or at the door of the slum resident—looking over one's shoulder. Putting a market price on body parts—even a fair one—exploits the desperation of the poor, turning their suffering into a medical opportunity. Asking the law to negotiate a fair price for a live human kidney goes against everything that contract theory stands for. When concepts such as individual agency and autonomy are invoked in defending the “right” to sell an organ, medical anthropologists suggest that certain “living” things are not alienable or proper candidates for commodification.

The problems multiply when the buyers and sellers are unrelated. In this situation, the sellers are likely to be extremely poor and trapped in life-threatening environments facing everyday risks to their survival, including exposure to urban violence, transportation- and work-related accidents, and infectious diseases that could compromise their single kidney. And when that ultimate “spare part” fails, kidney sellers often have no access to dialysis, let alone to organ transplantation. Moldova, which inadvertently supplied a great many desperate kidney sellers to affluent transplant tourists in Turkey, South Africa, and the United States, is today one of Europe's poorest nations. The country has only one public transplant unit and no capacity to guarantee dialysis to all those who may require it, least of all to rural men who fall into the hands of ruthless international kidney brokers.

Wouldn't a regulated system be better than the current state of racketeering in human kidneys? Perhaps, but how can a national government set a price on a healthy, but destitute, human being's body part without compromising essential democratic and ethical principles that guarantee the equal value of all human lives? Any national regulatory system would have to compete with global black markets that establish the value of human organs based on consumer-oriented prejudices. In today's kidney market, Asian kidneys are “worth less” than Middle Eastern kidneys and American kidneys worth more than European ones. The circulation of kidneys transcends national borders, and international markets will coexist and compete aggressively with any national, regulated systems. Surgeons whose primary responsibility is to provide care should not be advocates of paid self-mutilation by anonymous strangers even in the interest of saving lives.

Ethical solutions to the chronic scarcity of human organs are not always palatable to the public, but must be considered. Foremost among these are systems of educated, informed “presumed consent,” in which *all* citizens are assumed to be organ donors at brain death unless they have officially stipulated their refusal beforehand. This practice, which is widespread in parts of Europe, preserves the value of organ transplantation as a social good in which no one is included or excluded on the basis of their ability to pay.

While many individuals have benefited from the ability to get the organs they need through illegal circuits, the violence associated with kidney selling gives reason to pause. The division of the world into organ buyers and sellers is a medical, social, and moral tragedy of immense and not yet fully recognized proportions.
